# Elbow Dislocations: A Review Ranging from Soft Tissue Injuries to Complex Elbow Fracture Dislocations

**DOI:** 10.1155/2013/951397

**Published:** 2013-10-21

**Authors:** Carsten Englert, Johannes Zellner, Michael Koller, Michael Nerlich, Andreas Lenich

**Affiliations:** ^1^Department of Trauma Surgery, University Hospital Regensburg, Franz-Josef-Strauss-Allee 11, 93053 Regensburg, Germany; ^2^Centre for Clinical Studies, University Hospital Regensburg, Franz-Josef-Strauss-Allee 11, 93053 Regensburg, Germany; ^3^Department of Orthopedic Surgery, Ismaninger Strasse 22, 81675 Muenchen, Germany

## Abstract

This review on elbow dislocations describes ligament and bone injuries as well as the typical injury mechanisms and the main classifications of elbow dislocations. Current treatment concepts of simple, that is, stable, or complex unstable elbow dislocations are outlined by means of case reports. Special emphasis is put on injuries to the medial ulnar collateral ligament (MUCL) and on posttraumatic elbow stiffness.

## 1. Introduction

**Figure 1:**
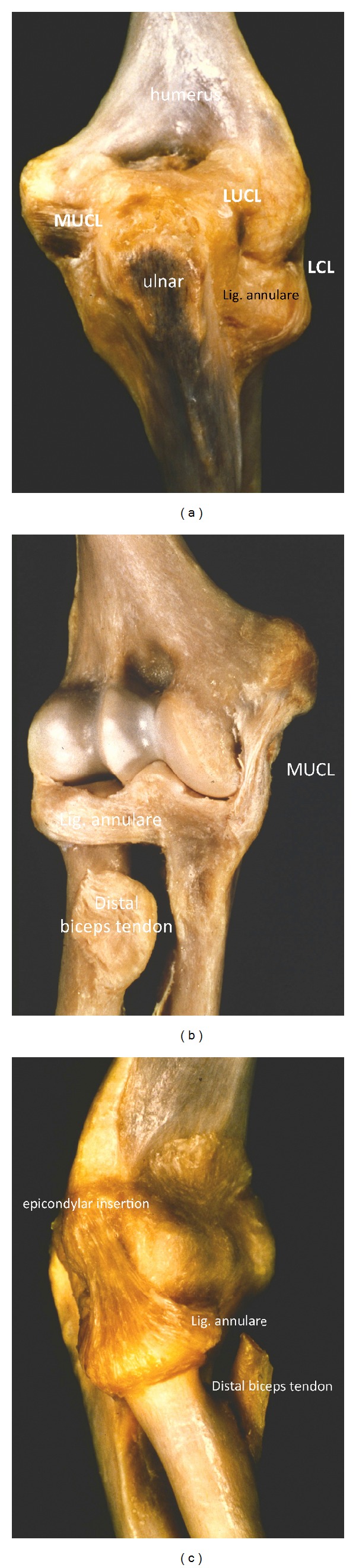
(a) Dorsal view to an anatomic elbow preparation. The posterior lateral ulnar collateral ligament (LUCL) and the posterior medial ulnar collateral ligament (MUCL) are visible. The posterior part of the ligamentum annulare with its insertion to the ulnar and the above lying distal part of the LCL can be seen. (b) Ventral view to an anatomic elbow preparation with demonstration of the anterior medial ulnar collateral ligament (aMUCL). The plane joint surface of the anteromedial facet of the olecranon can be seen, which has a buttress function in varus and valgus stress. (c) Lateral view to an anatomical elbow preparation with an illustration of the lateral collateral ligament complex (LCL) which is formed by the annular ring running to the radial epicondyle humeri.

Even simple elbow dislocations do not necessarily represent benign injuries. Anakwe showed in a retrospective trial with almost 180 patients that simple elbow dislocations caused considerable residual pain and stiffness in 62% and 56% of the patients, respectively [1]. According to Anakwe, elbow dislocations can be defined as simple if an elbow is stable when put through full range of motion after repositioning. Typically, simple elbow dislocations occur when a person falls onto an outstretched hand. Axial compression on the elbow in combination with supination and valgus stress primarily result in the rupture of the lateral ulnar collateral ligament (LUCL), which may cause posterolateral subluxation (Figure 1(a)). Extended elbow dislocations often involve severe ruptures with anterior and posterior capsule distractions followed by muscle injuries and, finally, by total elbow dislocation with rupture of the anterior MUCL (Figure 1(b)). This ligament tends to rupture in two stages; that is, the rupture of the posterior MUCL (Figure 1(a)) is followed by the rupture of the anteromedial bundle of the ligament complex [2, 3] (Figure 1(b)). This trauma mechanism has been described by O'Driscoll, who has also classified posttraumatic elbow instability according to the following five criteria: the articulations involved, the direction of the displacement, the degree of the displacement, the timing, and the presence or absence of associated fractures [3]. Fractures of a bony structure with a buttress function immediately result in elbow instability. The function of the medial ulnar collateral ligament complex and particularly its role in complex elbow dislocations has been investigated intensively. However, single injuries to the MUCL seem to be rare.

Overall, injury classification helps to understand the pathology of a trauma and aids in making decisions on the best form of treatment. However, not all of the wide range of treatment options available correspond to the criteria of evidence-based medicine [4]. Therefore, we present a treatment algorithm that is based on our clinical evidence with our preferred classification of elbow dislocations. Drawbacks and complications during the treatment of elbow dislocations are of particular interest, and special emphasis is placed on elbow stiffness. We also discuss new basic scientific aspects of the treatment of this complication and present first clinical results.

## 2. The Anatomy and Biomechanics of Ligaments and Bony Structures

The elbow represents one of the most stable joints in the human body. The close connection of the ulnohumeral and the capitulo-radial joints results in a high range of motion of the elbow with regard to extension and flexion as well as the pronation and supination of the forearm. Bony structures, the joint capsule, as well as the lateral and medial ulnar collateral ligaments allow direct motion control and high stability. The capitulo-radial joint compartment also contains ligament structures. The radial head is surrounded by the annular ligament that holds the radial head in place and makes rotational movements possible. The annular ligament encloses the radial head and inserts the ulna in dorsal and palmar directions (Figure 1(c)). The part of the annular ligament that lies above the LUCL runs into the lateral humeral epicondyle and forms the lateral collateral ligament (LCL). Bones and ligaments are considered static and primary stabilizing factors. Secondary stabilizing factors are joint-crossing muscles that additionally contribute to elbow stability [3].

**Figure 2:**
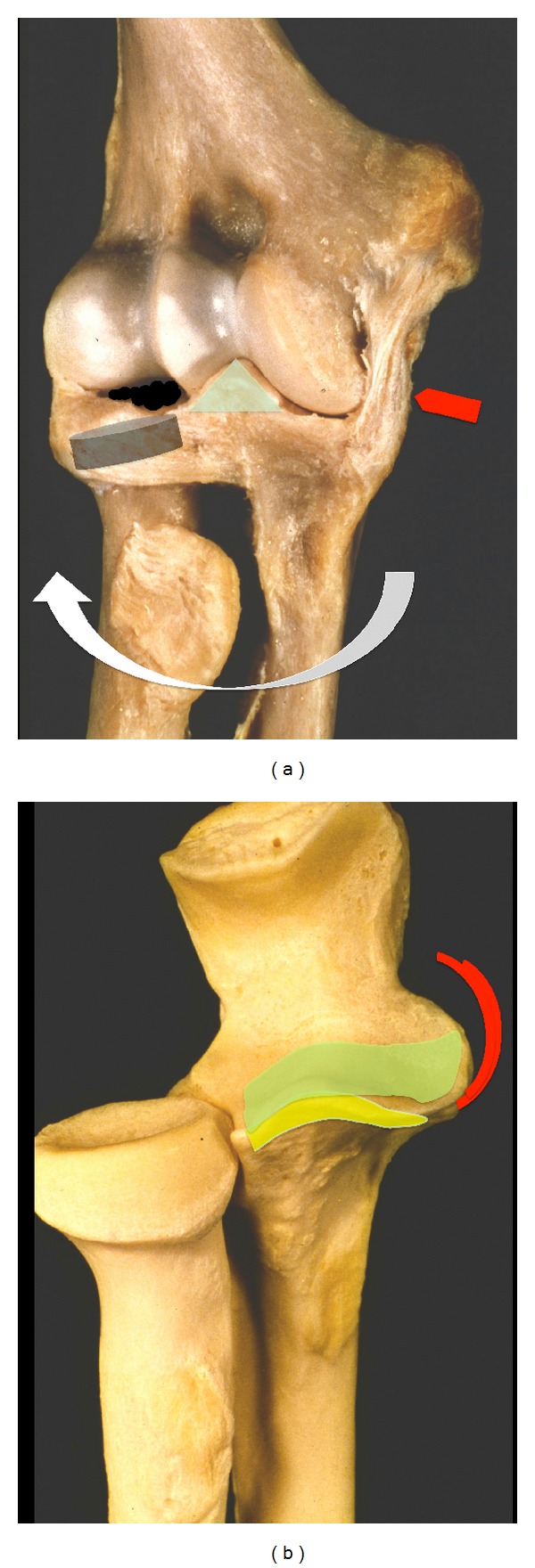
(a) The combined mechanical function of MUCL, anteromedial facet of the olecranon and radial head in valgus, and external rotational stability are demonstrated. (b) The yellow spot on the anatomical preparation illustrates types I and II coronoid fractures. Type III fractures of the olecranon (green spot) involve the anterior MUCL, which result in sudden angular and translational instability of the elbow between 30° and 60°.

The bony structures of the humeral trochlea sulcus and the connecting sigmoid notch of the olecranon have a buttress function and are the main stabilizing factor in varus and valgus stress and during rotational movements (Figure 1(b)). Caused by the semilunar surface of the capitulum humeri and the olecranon, the range of motion is limited in extension and flexion. Biomechanical trials have shown bony structures to be the main stabilizers of the elbow. 55% of varus stress is absorbed by bone compartments in full extension and even 75% at 90° of flexion. During varus stress, only minor support is given by capsular and ligamentous structures [5]. Valgus elbow stability depends on ligaments as well as on bony structures [6]. Experiments imitating coronoid fractures without any injuries to the ligaments as classified by Morrey have shown that resection of the radial head mainly influences valgus and external rotation, whereas coronoid deficiency results in varus laxity and posterior translation (Figure 2(a)). Overall, the radial head comprises approximately 40% of the stabilizing surface. 

Neither medial-oblique, lateral-oblique, nor even type II coronoid fractures caused any significant changes in elbow stability if the capsuloligamentous structures remained intact, even if the radial head had been removed. Type III fractures showed sudden angular and translational changes between 30° and 60° of elbow flexion, both in the presence or absence of the radial head (Figure 2(b)). The radial head is an important stabilizer during valgus and external rotation (Figure 2(a)), especially in type II or III coronoid fractures, whereas valgus and external rotational stability depend on the remaining total articular surface of the radial head. Posterior and proximal translations are influenced by the isolated articular surface involvement of the coronoid [7].

The MUCL is composed of three branches, the anterior, the posterior, and the oblique bundle, which is also commonly termed “transverse ligament.” Gross anatomical trials have shown that the anterior bundle is easily distinguished from the underlying joint capsule. The anterior bundle consists of two separate histological layers: the deeper layer is composed of collagen bundles contained within the capsule, and the shallower layer is a distinct ligamentous structure above the capsule. The anterior bundle originates from the anteroinferior edge of the medial humeral epicondyle and inserts on the sublime tubercle of the ulna. The posterior bundle originates from the posteroinferior aspect of the medial humeral epicondyle and has a broad insertion on the medial edge of the olecranon. The posterior bundle is not easily recognizable because it consists of a single layer of collagen bundles within the posteromedial aspect of the capsule. The transverse ligament varies in both size and gross appearance because it extends from the inferomedial margin of the coronoid process to the medial edge of the olecranon [6]. Biomechanical trials have shown that the MUCL predominantly contributes to the valgus stability of the elbow. The tension of the anterior and posterior bundles varies according to the degree of elbow flexion. In contrast to the anterior and posterior bundles, the transverse ligament is not important for elbow stability because it does not span the ulnohumeral joint [8]. The anterior bundle of the MUCL serves as the primary static stabilizer to valgus stress from 20° to 120° of elbow flexion [9]. The anterior bundle of the MUCL is more susceptible to valgus overload than the posterior bundle in extension or at a low flexion angle. The posterior bundle is more susceptible at higher flexion angles of the elbow. However, other authors have reported that the posterior bundle serves as a secondary stabilizer at high degrees of flexion [10]. Pollock et al. demonstrated that posterior bundle sectioning resulted in a 30% increase in maximum varus and valgus laxity and a 29% increase in maximum internal rotation during pronated active flexion. These findings suggest that the posterior bundle may be important in the late cocking phase of throwing [11].

The radial head is essential for the stability of the radial column of the elbow, particularly in elbow dislocation fractures involving injuries to the medial collateral ligament complex [3, 12]. During pronation and extension of the elbow, the maximal load is transmitted through the radial column with up to 60% of the load. Even a single radial head fracture with an intact medial collateral ligament complex results in up to 30% loss of resistance [13–15]. The posterolateral rotational stability depends on the lateral ulnohumeral ligament, the ligamentum annulare, as well as on the muscular structures [16]. 

## 3. Classification

### 3.1. Simple Elbow Dislocation from LUCL to MUCL

O'Driscoll postulated that elbows dislocate in 3 stages from the lateral to the medial side [3]. In simple posterior dislocations, the mechanism of injury can be thought of as a circle of soft tissue disruption that starts from the lateral side and progresses to the medial side. 

Stage 1 is characterized by the total disruption of the lateral ulnar collateral ligament and the partial or total disruption of the remaining lateral collateral ligament complex, resulting in posterolateral rotatory subluxation of an elbow that may reduce spontaneously. Patients suffer from pain when varus stress is applied to the elbow. 

Stage 2 includes the disruption of the anterior capsule that results in incomplete elbow dislocation in a posterolateral direction. X-rays may show a coronoid process perched on the humeral trochlea. Reduction is very easy and often unknowingly instigated by patients when bending the elbow. 

Stage 3 is divided into two subgroups:

- Describing the disruption of all soft tissues surrounding and including the posterior part of the medial collateral ligament except for the anterior bundle. This bundle forms the pivot around which the elbow dislocates in a posterior direction by way of a posterolateral rotatory mechanism;
- Being the complete disruption of the medial collateral ligament complex of the elbow [3].

Another model described by Ring and Jupiter [17] divides the stabilizing factors into anterior, posterior, medial, and lateral columns. The risk of chronic instability increases with the amount of columns injured. In this model, single MUCL rupture is viewed as a minor injury. No different grading exists for injuries to the MUCL in comparison to injuries to the LUCL as classified by O'Driscoll. 

### 3.2. Radial Head Fractures

Mason has classified radial head fractures into three types according to morph metric criteria [18]. This classification was extended by Johanson, who additionally described elbow dislocation:

Type I: 2-part radial head fractures without any or less than 2 mm of fragment dislocation,
Type II: 2-part radial head fractures with more than 2 mm fragment dislocation,
Type III: multiple fragment radial head fractures that are surgically reconstructable,
Type IV: Radial head fractures with additional ligamentous injury and dislocation.

### 3.3. Coronoid Process Fractures

Coronoid process fractures have been classified by Regan und Morrey [19] as well as by O'Driscoll et al. [2]. Regan and Morrey simplified matters by dividing coronoid fractures in the sagittal plane into two groups, above 50% or below 50% of the height measured from the tip to the baseline. O'Driscoll's classification takes into account that coronoid process injuries are shear stress fractures that involve the ligamentous insertion of the medial ulnar collateral ligament complex (Figure 2(b)). Ulna stress leads to an avulsion fracture of the coronoid process by the anteromedial bundle of the medial ulnar collateral ligament (MUCL).

### 3.4. Terrible Triad Injury

Injury mechanisms with compressive force to the radial head and a fracture line in the radial neck should draw attention to the lateral ulnar collateral ligaments (LUCL). The combination of coronoid and radial head fractures with rupture of the (LUCL) was first described by Hotchkiss [20] and has been termed “terrible triad injury.” According to the stability criteria and column theory established by Ring and Jupiter [17], at least two stabilizing columns (anterior and lateral) are lost in such injuries. Because of the coronoid fracture, the medial column may also be involved. If the fracture line crosses the coronoid base, in which the anteromedial bundle of the (MUCL) inserts, the medial column is also lost. Resection of the fractured radial head is likely to destabilize the elbow, which increases the risk of further dislocation and posttraumatic instability. 

### 3.5. Fracture Dislocations of the Elbow

Elbow dislocations are not only characterized according to the morphological criteria of the radial head and the coronoid, but mainly according to the direction of dislocation, namely, anterior, posterior, and divergent. The position of the forearm is described to the upper arm. Posterior dislocations are the most frequent, and anterior dislocations are the least common form. Anterior elbow dislocations always involve fractures of the olecranon. Divergent elbow dislocations separating the radius and the ulna occur in high-impact trauma and include rupture of the membrana interossea, ligamentum annulare, and often rupture of the distal radioulnar joint. 

### 3.6. Anterior or Transolecranon Fracture Dislocations

This complex injury occurs after a direct high-energy blow to the posterior aspect of the forearm with the elbow in 90° of flexion [21]. The forearm dislocates in anterior direction in relation to the incisura trochlearis in contrast to the anterior Monteggia dislocation. Distal of the ulna fracture line, the membrana interossea, and the distal radio-ulnar joint remain intact. Anterior Monteggia dislocations involve additional injuries to the membrana interossea and the distal radio-ulnar joint [22].

### 3.7. Posterior Monteggia Fracture Dislocations

This injury occurs after high-energy traumas and involves a multifragmentary fracture of the proximal ulna. Triangular or quadrangular fragments are frequent and typically involve the coronoid process. Usually, the radial head becomes fractured and dislocated dorsally to the distal ulna fragment. The lateral ligament complex may be torn, but the medial ligament remains intact.

In elbow dislocation fractures, it is most important to reconstruct the ulnohumeral joint, particularly fragments of the coronoid base. The anteromedial bundle of the medial collateral ligament inserts on the coronoid base, stabilizing the joint during valgus stress, which represents an important part of the stabilizing medial column. Loss of the medial ulnar collateral ligament in the context of a fractured coronoid be treated surgically, for example, by splitting the flexor muscles [23]. Complex fractures involving instability after internal osteosynthesis and ligament reconstruction can be treated with the additional application of a hinged fixator that can be applied with a limited range of motion in very difficult cases [24–27].

## 4. Diagnostics

Patient questionnaires on elbow dislocations should include topics, such as trauma mechanism, impact, and posttraumatic neurological sensations. Clinical examinations should start distally from the injured joint and include the checking of vascular, sensory, and muscular functions. Two-dimensional X-ray images should be taken before and after repositioning maneuvers and should include the radial head and the olecranon. CT scans may be indicated in case of inconclusive X-ray results or if surgery is required.

Ligament elbow injuries are difficult to detect on X-ray images. For example, initial subluxation of the elbow with rupture of the (LUCL) may lead to persistent posteroradial instability [28]. Therefore, an MRI should be conducted in inconclusive cases or in patients with clinical symptoms persisting for more than 10 days. 

**Figure 3:**
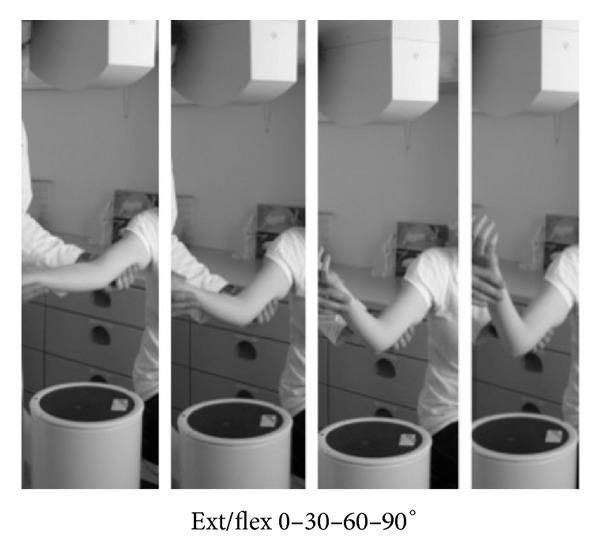
Examination of the elbow joint under intensifier during 0-30-60-90° of varus and valgus stresses.

We recommend the following procedure for testing for elbow ligamentous instability. The patient's arm should be positioned under the radiograph intensifier as shown in Figure 3. Humero-ulnar joints should be examined during valgus and varus stress tests at the flexion angles of 0°-30°-60°-90° under the radiograph intensifier (Figure 3). Instabilities become visible by a widened joint gap that can be documented by a printout. A standardized documentation may allow the specification of the injured ligaments (Table 1).

## 5. Therapeutic Strategies for Elbow Dislocations Depend on the Injury Classification

The main goal in the treatment of elbow injuries is mobilization within the first three weeks after trauma. Unfortunately, hardly any evidence-based data are available in this respect [4]. Most of the relevant literature reports state that clinical outcomes decrease with increasing time until the immobilization of the elbow after trauma. Based on the classification established by O'Driscoll [29], Bell [30] presented an algorithm for simple elbow dislocations, which categorized three stages of instability between athletes and nonathletes. Because of the lack of sufficient evidence-based data, we developed our own posttrauma concept for simple elbow dislocations that was based on biomechanical findings and our own experience. Choice of therapy, that is, surgical or conservative treatment, depends on the individual instability criteria present. Severe joint and ligament reconstructions with persisting instability require elbow bridging with a hinged fixator with or without limited extension of the elbow to retain stability and joint movement [27], which will be explained in the following.

### 5.1. Single Capsular and Ligamentous Injuries

In elbow dislocations, single ligamentous or capsular injuries are rare [31]. Patients with stages 1 and 2 of elbow dislocations may be treated by distraction and flexion, whereas patients with severely dislocated elbows at stage 3 should only be treated under anesthesia. For all patients, stability tests should be carried out immediately. Patients suffering from pain should also be treated under anesthesia and with technical support by fluoroscopic guidance (Figure 3). 

Once an elbow is stabile after reduction, a plaster cast is applied for 3 to 5 days to maintain the forearm in supination and the elbow flexed at 90°. Lymphatic drainage and a muscle pump should be applied. According to the guidelines developed by the German Association for Evidence-Based Medicine (Arbeitsgemeinschaft der Wissenschaftlichen Medizinischen Fachgesellschaften e.V., AWMF), thrombosis prophylaxis is not necessary [32]. It is mandatory, however, that patients are clinically reevaluated within 7 days after the trauma. To relieve the injured ligament complex (partially or completely torn LUCL and MUCL), a splinted brace with limited extension or flexion is applied for 6 weeks. The range of motion needs to be adjusted according to the injured ligament. For example, the torn LUCL could be set at 0-30-110° for extension and flexion in weeks 1 and 2, at 0-20-120° in weeks 3 and 4, and at 0-10-130° in weeks 5 and 6. After the 6th week, free range of motion with lightweight should be possible. The range of motion is then expanded by 10° per week according to a patient's pain symptoms. Physiotherapists can monitor the healing process with its inflammatory reaction. However, elbow injuries may result in stiffness as well as in instability. Both conditions have to be identified during both surgical and conservative treatments, and therapeutic strategies have to be adjusted according to possible complications during the healing process. 

If an elbow is unstable in extension, it can rarely be stabilized by a plaster cast at 90° of flexion. In such cases, the cast has to be applied at 110° of flexion because such an angle will reduce even most complex elbow dislocations (stage 3). The tension of the triceps tendon and the bony formation of the humeral groove to the proximal ulnar will reduce and stabilize the elbow in an anatomical position. Sometimes, interposition of in the capsule, ligaments, or muscles or even chondral fragments may block the repositioning of the elbow; such patients require immediate surgery within 24 hours. Unstable elbows due to a torn capsule and ligament structures need to be stabilized by the open reconstruction of the ligament structures and/or by applying a hinged fixator. There are a lot of discussions about which intervention is best, ligament reconstruction alone or a hinged fixator without or with ligament augmentation. Some authors prefer ligament reconstruction [3, 33], others ligament reconstruction with a hinged fixator [31], and still others a hinged fixator alone [34]. 

### 5.2. Elbow Dislocation Starting from the Radial Column

**Figure 4:**
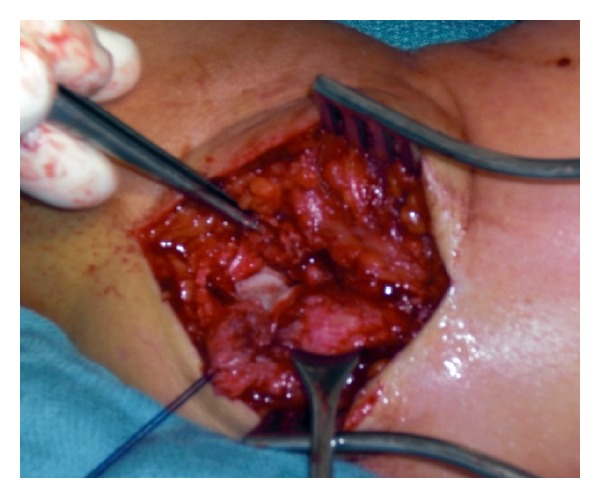
Isolated rupture of the lateral ulnar collateral ligament (LUCL) resulting in persistent posterolateral rotational instability (PLRI) in a patient who works as an anesthesiologist. She was not able to retract the speculum after trauma. The picture illustrates the intraoperative reconstruction of the proximal torn LUCL.

Radial head injuries should be handled with care, and possible injuries to the lateral ligament complex should be excluded. Some authors have suggested that the choice of treatment of singular radial head injuries should depend on the extent of fragment dislocation [35, 36]. In simple elbow dislocations, the radial column is often combined with a radial head injury type Mason II and rupture of the lateral ulnar collateral ligament (LUCL). This common complication often results in persistent posteroradial instability and should be addressed by reconstructing the radial head and by augmenting the ruptured ligament complex [12] (Figure 4). Biomechanical investigations have shown that absence of the radial head induce rotatory laxity of 145% compared to intact elbow joints. Absence of the radial head and the coronoid process results in the deviation and dislocation of the joint, regardless of its collateral ligament status [37]. Biomechanical trials have shown that the lateral collateral ligament is the primary stabilizer to external rotation and posterolateral stabilization (Figure 2(a)). Reconstruction of the LUCL alone, even without the radial head, is beneficial. 

Because of the shear forces caused by a dislocation, the capitulum humeri should be checked for osteochondral defects, even if none were detected on MRI. 

### 5.3. Terrible Triad Injury

The insufficiency of the available data and the manifold posttraumatic complications in elbow injuries have resulted in a wide range of suggestions on how to treat terrible triad injuries [38, 39]. Although some authors have suggested conservative treatment, terrible triad injuries always result in elbow instability and thus always necessitate surgery in our opinion.

#### 5.3.1. Radial Column in Terrible Triad Injuries

50% of terrible triad elbow injuries are marked by a complete rupture of the radial capsuloligament complex combined with injuries to the osseous and articular lignment [21]. Because of the high risk of redislocation, chronic instability, and posttraumatic arthritis, surgery is justified. Fractured radial heads and the torn ligaments should be restored to rebuild the radial column. The elbow prefers to bend into the valgus position. Without any tension on the ulnar complex, the radial head is more loaded by the deviation of the elbow. Therefore, reconstruction of the radial head is necessary, which can be very difficult in some cases. Even the option of reconstructing the radial head on a table followed by reinsertion in situ has been suggested to avoid a radial head prosthesis. However, if there is no alternative, a radial head prosthesis is an adequate method for restoring the radial bony column. To address posterolateral instability due to the rupture of the radial humero-ulnar ligament, the ligament complex should be reattached by suture or with a suture anchor. Ligaments are mainly ruptured in the proximal third and can easily be reattached by transosseous sutures. 

#### 5.3.2. Coronoid Process Fractures and the Ulnar Ligament Complex

The classification of fractured coronoids by Morrey and Regan can be expanded by the O'Driscoll classification, which provides information about the involvement of the medial column and ligament complex (Figure 2(b)). In fractures of the coronoid facet of the ulnohumeral joint (type III), the bony stabilizing column on the medial side is lost and has to be restored. Such restoration can be done by an anteromedial approach, that is, by splitting the flexor muscles [40]. Small coronoid shear fractures (types I-II) with a ruptured anterior capsule and muscle brachialis can be reattached by suturing the anterior capsule to the proximal ulna through drill holes from a radial approach. By this approach, ruptured lateral ligament complex can be easily stabilized by transosseous suture. The coronoid can also be addressed by a posterior approach by releasing the flexor muscle group or by an anteromedial approach by splitting the flexor muscle group with readaptation of the anteromedial capsula and medial ulnar collateral ligament complex (anterior ulnohumeral bundle of MUCL) [3]. If the lateral ulnar collateral ligament (LUCL) has to be addressed, the coronoid tip can be fixed by a lateral approach (Kochers approach).

Readapting the medial ulnar collateral ligament (MUCL) without the coronoid process is rarely necessary. The buttress function of the lateral column is restored after rebuilding the radial column by osteosynthesis of the radial head and suture of the LUCL. Only if the restoration of the radial column is fragile or instable with persistent valgus stress to the elbow should the ulna ligament complex be restored or a hinged external fixator be applied with a limited range of motion to 0–40–140° for 3 weeks [27].

### 5.4. Elbow Dislocations Affecting the Medial Column

**Figure 5:**
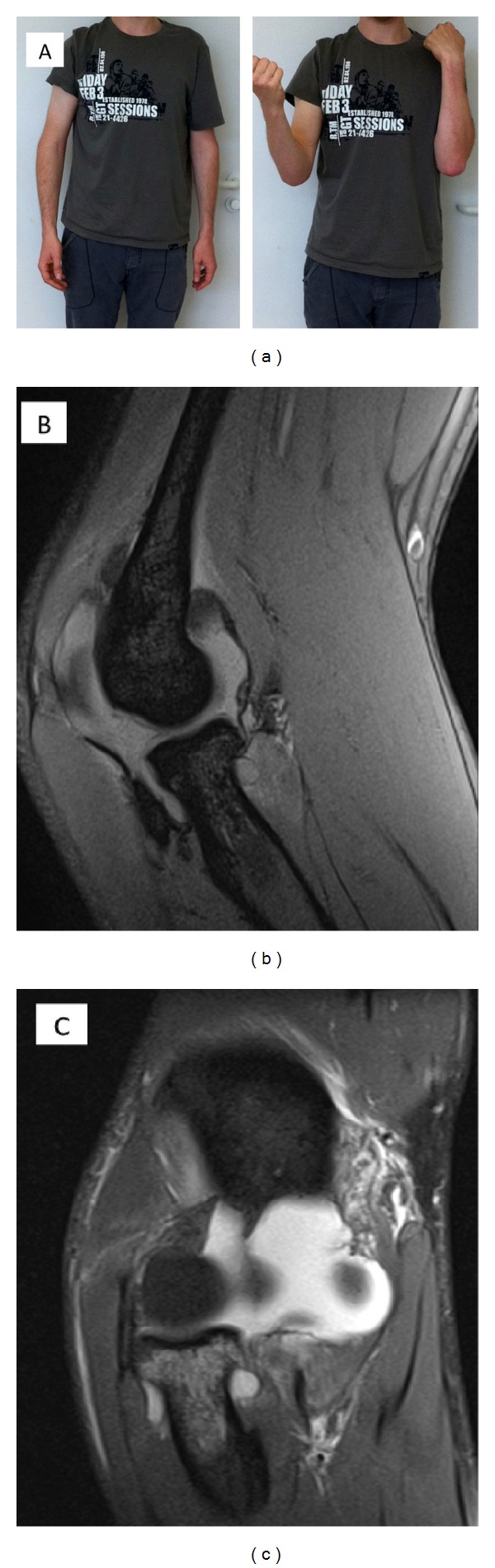
(a) Isolated rupture of the anterior medial ulnar collateral ligament (aMUCL) by valgus stress trauma. The patient was typically suffering from a limited range of motion in flexion. Full range of motion with a stable elbow was restored with conservative treatment 10 weeks after trauma. (b) MRI proofed isolated aMUCL rupture of the photographed patient. The sagittal plane illustrated an inflammatory reaction. (c) The frontal plane demonstrated the rupture of the MUCL complex from its proximal insertion on the medial epicondyle humeri.

Most of the knowledge on single MUCL lesions has been gained from examining athletes such as javelin throwers or handball players [41]. Chronic instability requires surgery including ligament augmentation [42]. In our experience, acute trauma patients with a valgus stress injury and a consecutive single MUCL lesion suffer from limited range of motion (Figure 5(a)). Two-dimensional X-ray images of the elbow often show no fractures with regular alignment. MRI illustrates in such cases single MUCL injury (Figures 5(b)-5(c)). However, patients unable to bend their elbow are usually in pain if they try to do so. Patients tend to extend their elbow and reject any flexion above 80°, which is a strong clinical marker for MUCL distortion or rupture. Bracing the elbow with a limited range of motion in flexion will lead to a pain-free stable elbow within 2 to 4 weeks or, in severe cases, up to 8 weeks. 

## 6. Complications in Elbow Dislocations and Dislocation Fractures

### 6.1. Chronic Instability

Pain and persistent instability have to be observed and, in individual cases, stabilized by ligament complex augmentation [43, 44]. Sometimes, elbow dislocations categorized as stage 1 and stage 2 are underestimated. Insufficient clinical and radiological examinations may classify an injury as a distortion, and the patient is discharged without any further treatment. Ruptured collateral ligaments result in hyper-mobility of the elbow joint; this condition leads to the very painful overcompensation of the extensor muscle group [45]. Elongation of the scar tissue of the ruptured LUCL complex requires a ligament plastic. Several methods have been described for this procedure, such as the transplantation of a biceps tendon through the coronoid process or reinforcement of the collateral ligaments [46] or the reconstruction of the collateral ligament complex by autograft transplantation of triceps tendon [44, 47] or by a gracilis graft [48].

In elbow fracture dislocations, surgeons aim at achieving anatomical reduction and stabilization of the elbow joint [49]. The medial facet of the coronoid to the humeral trochlear is identified as a key factor for the medial stability of the elbow, and intensive reconstruction maneuvers are indicated to regain stability of the elbow joint if the medial facet of the coronoid is lost [50, 51]. Milch's and Wainwright's presented a bone block procedure, which has a mechanical effect on the coronoid process to prevent its disengagement under the trochlear by an anterior approach [46]. Furthermore, osteochondral autografts have been tried for rebuilding the lost medial coronoid facet, but only with minor success [52]. 

### 6.2. Elbow Stiffness and Physiotherapy

In posttraumatic care, elbow arthrofibrosis and heterotopic ossification are common complications that depend on the severity of an injury [1, 53]. Arthrofibrosis frequently occurs after elbow dislocation leading to a median extension loss of 8° after one year [54]. In a trial by Protzman et al., almost 50% of patients developed heterotopic ossifications of the torn ligaments. Other authors reported an incidence of posttraumatic heterotopic ossification between 1.6% and 56%. In elbow fracture dislocations, elbow stiffness and heterotopic ossification affect up to 20% of the patients [55, 56]. Concurrent elbow and radial head injuries raise this incidence rate to almost 90%, and even patients with an isolated injury to the radial head suffer elbow stiffness in 5 to 10% [57, 58]. Duerig reported that elbow dislocations are rarely unstable in the absence of an associated articular fracture. Most posterior elbow dislocations are stable and would, thus, benefit from active exercises and functional use of the arm within 2 weeks after the injury [59]. Mehlhoff et al. suggested that early mobilization is paramount for avoiding arthrofibrosis in elbow dislocations and is, thus, the key factor in posttraumatic care. However, in a followup of 52 patients, the same authors reported 24 patients suffering from persistent pain and 34 patients with a limited range of motion, which is a rather questionable result [60]. Yet, early mobilization has been suggested by the AAOS for simple and complex elbow dislocations [61]. This elbow mobilization program allows controlled movements without putting strain on injured structures. However, little data is available on early motion therapy. 

**Figure 6:**
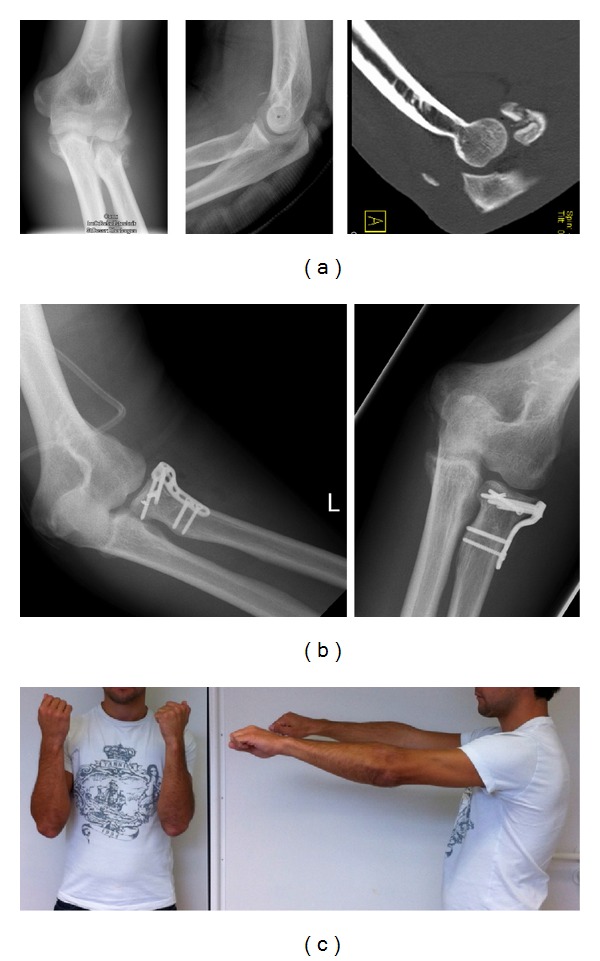
(a) X-rays illustrating radial head multifragmentary fracture. One slice of the CT scan proves the bony tendon tear of the triceps by a young sports student, who was suffering from an elbow dislocation. (b) Postoperative X-rays demonstrating radial head reconstruction and stabilization. The bony tear of the triceps tendon was sutured to the olecranon tip. (c) Forty-eight weeks after trauma, the young athlete was not able to straighten the left elbow. Full range of motion was restored within 6 weeks by applying oral cortisone therapy with Prednisolon 5 mg tablets in decreasing dosage as described in Table 2.

From a basic research view, inflammatory reactions of the healing process are responsible for capsulitis and shrinkage of the capsula by SMA myofibroblasts [62]. A torn capsula will inflame and develop a kind of capsular adhesive during the healing process. Scar tissue formed by fibrocytes and myocytes may impede the desired healing process, particularly if an elbow is immobilized for 3 weeks after the trauma. According to our experience, elbows will also get stiff when immobilized for more than 3 weeks after the trauma. However, capsular fibrosis can be stopped and even reduced again by the oral administration of cortisol and spironolactone [63–65] (Table 2, Figures 6(a)–6(c)). Temporary elbow stiffness is a normal occurrence and should be treated with pain-free long-lasting flexion and extension exercises over a long period of time. In severe cases, cortisol and spironolactone may be administered additionally. 

### 6.3. Heterotopic Ossification in Elbow Dislocations

Heterotopic ossification (HO) of the elbow is a common posttraumatic occurrence resulting in limited range of motion [66]. The role of HO in elbow stiffness has to be analyzed by CT scans with three-dimensional volume rendering, which helps to analyze bony impingement areas. Arthroscopy is a common approach for releasing HO and regaining better range of motion [67–69]. Risk factors for HO are the time elapsed between trauma and surgery as well as the number of days of immobilization after surgery [70]. Anti-inflammatory drugs, such as indometacin and radiation therapy, have been suggested as part of the operative treatment of HO [69]. 

## 7. Conclusion

1. Elbow dislocation is a severe injury leading to disability. Initial diagnostics should include instability tests under fluoroscopic guidance.
2. Patients with severe elbow instability require an MRI or a CT scan.
3. In case of instability after repositioning, a plaster cast should be applied at 110° of flexion, and secondary surgical stabilization needs to be achieved within 5 days.
4. Elbows that have been fluoroscopically proven to be laterally instable or medially stable have to be further investigated for posterolateral instability, which requires surgical reconstruction of the LUCL complex.
5. Fluoroscopically proven medial instability and a laterally stable band complex can be treated conservatively with a plaster cast at 60° of flexion und light supination of the forearm.
6. Elbow distortions causing persisting pain should be examined with an MRI to check for possible band or cartilage injuries.
7. Stiffness and heterotopic ossifications after elbow dislocation are common occurrences and should be treated by controlled early mobilization in braces with limited range of motion and, in severe cases, by oral steroid medication.

## Figures and Tables

**Table 1:** Classification of elbow instability and labeling of injured ligaments.

Direction of instability		
Varus		Valgus
LCL + LUCL	0°	aMUCL
LCL > LUCL	30°	aMUCL > pMUCL
LCL ≥ LUCL	60°	aMUCL < MUCL (pMUCL)
LUCL	90°	MUCL (PMCL)

Overextension	Ventral capsule + aMUCL + LCL	

**Table 2:** Oral cortisone drug medication in stiff shoulder or stiff elbow therapy.

Period of therapy	Dosage of oral cortisone therapy
1–5	40 mg per day
6–10	30 mg per day
11–15	20 mg per day
16–20	10 mg per day
